# 

**Published:** 1890-05-10

**Authors:** 


### THE HOSPITAL

A WEEKLY JOURNAL OF
Science, Medicine, Nursing and Philanthropy.

SATURDAY, MAY 10th, 1890.

**A Standard of Sanity** - 73

**Words of Consolation.**—Patience in Sickness - 74

**Working Women in Large Towns.**—II. Street Workers - 75

**Do the Sick Fear Death?** - 76

**The Doctor's Pulpit.**—A Doctor "on the Stump" - 77

**Watching the Insane by Reflecting Shafts** - 78

**Everybody's Page** - 79

**Notes and News** - 80

**Annotations.**—What is an Expert?—Doctors and Drink—Prescriptions in the Mother Tongue - 82

**The Practitioner's Mirror and Retrospect:**

Medicine—Addison's Disease - 83

Surgery—Pelvic Inflammation - 83

The Practitioner's Bookshelf.—Applied Anatomy, Medical, Surgical, and Operative - 84

**Vaccination in Italy** - 84

**The Hospital Workshop.**—XXVI. Stroud General Hospital 85

**Passing Topics.**—Retiring Allowances to Charity Officials - 88

**Scraps and Gleanings** - 88

**The Students' Column** - 88

### EXTRA SUPPLEMENT—THE NURSING MIRROR.

**En Passant.**—Dramatic Performance—Redditch Nursing Association—District Nursing at Ballymena—Nurses and Infection—Short Items—Liverpool Nurses—Belfast Nurses - xxi

**Nursing Lectures.** By Percy Lewis, M.D. XI.—Nervous Diseases - xxii

**The Private Nurses' Co-operation** - xxii

**Everybody's Opinion.**—The Junius S. Morgan Memorial—Uniforms for June - xxiii

**The "First Thousand"** - xxiii

**Examination Questions** - xxiii

**Presentations—Notes and Queries** - xxiv

**Appointments—Vacancies** - xxiv

**Amusements and Relaxation** - xxiv

